# A murine model to evaluate immunotherapy effectiveness for human Fanconi anemia-mutated acute myeloid leukemia

**DOI:** 10.1371/journal.pone.0292375

**Published:** 2024-01-30

**Authors:** Tingting Huang, Bernice Leung, Yuyang Huang, Laura Price, Jiang Gui, Bonnie W. Lau

**Affiliations:** 1 Dartmouth Health Cancer Center, Department of Pediatrics, Geisel School of Medicine at Dartmouth College, Lebanon, NH, United States of America; 2 Department of Biomedical Data Science, Geisel School of Medicine, Lebanon, NH, United States of America; University of Missouri, UNITED STATES

## Abstract

Fanconi anemia (FA)-mutated acute myeloid leukemia (AML) is a secondary AML with very poor prognosis and limited therapeutic options due to increased sensitivity to DNA-damaging agents. PD-1 immune checkpoint inhibitors upregulate T-cell killing of cancer cells and is a class of promising treatment for FA-AML. Here, we developed a novel FA-AML murine model that allows the study of human AML with a humanized immune system in order to investigate immunotherapeutic treatments *in vivo*. FA-AML1 cells and non-FA-mutated Kasumi-1 cells were injected into 8–10 week old NSG mice. Once leukemic engraftment was confirmed by HLA-DR expression in the peripheral blood, human peripheral blood mononuclear cells (hPBMCs) were injected into the mice. One week post-hPBMCs injection, Nivolumab (PD-1 inhibitor) or PBS vehicle control was administered to the mice bi-weekly. In our Nivolumab treated mice, FA-AML1, but not Kasumi-1-engrafted mice, had significantly prolonged overall survival. Both FA-AML1 and Kasumi-1 engrafted mice had decreased spleen weights. Higher leukemic infiltration into vital organs was observed in FA-AML1 engrafted mice compared to Kasumi-1 engrafted mice. In conclusion, our novel humanized murine model of FA-mutated AML is an attractive tool for supporting further studies and clinical trials using PD-1 inhibitors to treat FA-mutated AML.

## Introduction

Acute myeloid leukemia (AML) is the most common cancer that arises in patients with Fanconi anemia (FA), an inherited bone marrow failure syndrome with germline DNA damage repair defects in the FA/BRCA pathway, and significantly elevated cancer risks [1, 2]. FA-AML portends poor prognosis due to tumor resistance to standard AML treatment, high risk of AML relapse, and increased risk of treatment-related toxicities. It is imperative to find new therapies for FA-AML that are more effective and less toxic. We have established a novel humanized murine model to test immunotherapy for FA-AML. Traditionally, FA-AML biology has been studied using murine leukemia in transgenic models of FA. However, the FA transgenic mice do not naturally develop FA-AML [3, 4]. Rather, the mice require exposure to DNA damaging agents such as mitomycin C or irradiation in order to develop FA-AML [4]. Therefore, FA-AML being studied in transgenic models potentially differs from the human disease observed clinically.

With the development of humanized murine models, human cancers can be studied in immunocompromised mice, allowing for engraftment of human tumor cells for *in vivo* studies [5, 6]. Humanized murine models can be particularly useful for studying rare diseases such as FA, since getting a significant number of FA patient samples for research is challenging. In 2011, a group published work showing successful engraftment of patient derived FA-AML [7]; however, there was no engraftment of a human immune system in the model. We aim to establish a humanized murine model that includes not only human FA-AML, but also human immune cells to properly study immunotherapy and other immune-mediated therapies for FA-AML. For our studies we compared humanized mice engrafted with FA-AML to humanized mice engrafted with a non-FA mutated AML.

Functionally, we demonstrate that our tumor- and immune cell-humanized murine model works for testing immune checkpoint inhibitor (ICI) therapy in AML. ICI has been successfully used to treat many solid tumors such as colon cancer, melanoma, breast cancer, ovarian cancer and lung cancer [8–10]. There is now evidence that ICI therapy, particularly PD-1 inhibition, can prevent AML relapse in adult and it is currently undergoing clinical trials for relapsed/refractory AML in children in combination with 5-azacytidine [11–15]. We show that PD-1 inhibition can be effective for FA-AML using our novel humanized murine model. Since PD-1 inhibition is especially effective for colon cancers with mismatch repair deficiencies, we hypothesized that FA-mutated AML with DNA damage repair defect in the FA/BRCA pathway would similarly respond more effectively to PD-1 inhibition than non-FA mutated AML.

## Materials and methods

### Cells

The Fanconi Anemia (FA) Acute Myeloid Leukemia (FA-AML1) cell line and U-937 cell line were obtained from Dr. Stefan Meyer (The University of Manchester, UK) [16]. Conditioned medium from U-937 cells contains various cytokines, chemokines and growth factors used to supplement AML cell growth. FA-AML1 cells were grown in RPMI 1640 (Corning) supplemented with 20% Fetal Bovine Serum (FBS) (Cytiva), 1% penicillin/streptomycin (Sigma), 10% U-937 conditioned media and 10 ng/mL recombinant human interleukin-3 (rhIL-3, PeproTech). Kasumi-1 cells were purchased from ATCC and grown in RPMI 1640 supplemented with 20% FBS, 1% penicillin/streptomycin and 10% U-937 conditioned media [17]. U-937 cells were grown in RPMI-1640 supplemented with 10% FBS. All cells were cultured in a humidified 5% CO_2_ incubator at 37°C and tested negative for mycoplasma. All cell lines have been authenticated by STR profiling.

### Mice

NSG (NOD.Cg-Prkdcscid Il2rgtm1Wjl/SzJ) mice were obtained from Dr. Steven N. Fiering (Dartmouth College, NH) and maintained in a temperature-controlled research animal facility. Animals had free access to food and water and were housed in individually ventilated cages on a 12-hour light–dark cycle. Kasumi-1 and FA-AML1 cells were washed and re-suspended in PBS. 5 x 10^6^ cells were injected into non-irradiated 8–10 weeks old male or female NSG mice through intravenous administration [18]. The mice were in 2.5% isoflurane during intravenous trail vein injection and placed on a heating pad for anesthetic recovery. Once leukemia engraftment was confirmed, 1 x 10^6^ human peripheral blood mononuclear cells (ATCC, PCS-800-011) were injected into the mice intravenously. hPBMC engraftment was confirmed one week post-hPBMC injection, and Nivolumab treatment was initiated.Mice were treated with PBS or Nivolumab (Selleckchem, A2002) bi-weekly after randomization of 8 mice per group until death. Signs of pain and distress were monitored daily by veterinary technicians, and animals were euthanized with carbon dioxide exposure followed by cervical dislocation. After mouse death, the lung/liver/spleen/kidneys were collected by necropsy and sent for H&E staining. Organs were only collected from mice dead for no more than one day as organ swelling was observed in some post-mortem animals which may cause weight or histology variations. All experimental procedures conducted in this study were approved by the Institutional Animal Care and Use Committee (IACUC protocol #00002245 by Kirk Maurer, DVM) of Dartmouth College.

### Cell viability assay

Cell viability was measured using MTT assays. 10,000 cells/well were seeded onto 96-well plates. FA-AML1 and Kasumi-1 cells were treated with a gradient concentration of Nivolumab for 48 hours. Cells were stained with 50μg of MTT (Invitrogen) and acidified ispropanol was added to dissolve the formazan substrate. Absorbances were measured at 540 nm using the SpectraMax i3x (Molecular Devices).

### Flow cytometry

Engraftment of human Kasumi-1 and FA-AML1 cells was verified by flow cytometric analysis of peripheral blood (PB). Briefly, 100 μL of blood was collected from the submandibular vein every week from 8 weeks after cell injection. Following lysis of red blood cells by RBC lysis buffer according to manufacturer’s protocol (Biolegend, 420301), the blood was washed twice by Cell Staining Buffer (Biolegend, 420201), then stained with anti-human HLA-DR (BD, 560744). Human HLA-DR positivity (>1% in PB) was used to confirm Kasumi-1 and FA-AML1 engraftment [19]. Engraftment of hPBMCs was determined by flow cytometric analysis. Following peripheral blood collection and RBC lysis, the cells were stained with anti-human CD3 (Biolegend, 317306). Cell surface level PD-L1 was measured after stimulating Kasumi-1 and FA-AML1 with 10 ng/mL IFNγ (R&D Systems, 285-IF) for 24 hours and staining with anti-human PD-L1 (Biolegend, 374514). Flow cytometry was carried out in the Immune Monitoring and Flow Cytometry Shared Resource at the Dartmouth Cancer Center, with Cancer Center Support Grant 5P30 CA023108-41.

### RNA extraction and quantitative reverse-transcription polymerase chain reaction

Total RNA was extracted using TRIzol (Invitrogen) and RNeasy Mini Kit (Qiagen) following manufacturer’s protocol. Briefly, RNA precipitated from phenol/chloroform TRIzol extraction was transferred to RNeasy mini spin columns for RNA clean up. cDNA synthesis was carried out using SuperScript™ III First-Strand Synthesis System (Invitrogen, 18080051). Expression of PD-1 and PD-L1 were examined by CFX96 Touch Real-Time PCR Detection System (Bio-Rad) using PowerUp™ SYBR™ Green Master Mix (Applied Biosystems). Relative expression was calculated using 2^-ΔCq^ with reference to β-actin expression.

### Histology

Mouse spleen was collected with necropsy after mouse death and weighed. All mouse tissues were fixed in 10% formalin, paraffin-embedded, sectioned (5 μm sections), and stained with hematoxylin and eosin (H&E), or antibody-stained for human CD33, CD8 or cleaved caspase-3. Histopathology for AML in the organ sections was identified for blast morphology (increased nuclear:cytoplasmic ratio and dense chromatin, increased nuclear blue stain). Histology was carried out in the Pathology Shared Resource at the Dartmouth Cancer Center, with NCI Cancer Center Support Grant 5P30CA023108-37. Slides were imaged using the Olympus IX73 inverted microscope on a 20x objective.

### Statistical analysis

All statistical analyses were performed using GraphPad Prism v9. Descriptive analysis was used to characterize groups. Log-rank test was used to compare survival between groups. Unpaired, two-tailed Student’s t test was used to determine statistical significance of differences. P < 0.05 was considered significant. For spleen weights [Fig 3C and 3D] we observed outliers from the dot plot while comparing group x with group y. It is possible because necropsy was performed from spleens collected at various times after death, causing variation with organ desiccation or organ swelling post-mortem, so we are limiting to spleen weights from mice that were not dead for more than 1 day. We define outlier as any observation which is outside of the range of mean +/- 2*SD (standard deviation) estimated by the rest of the observations. As a result, we have removed 1 observation from group x and y respectively. We used T-test to compare the means of the two groups afterwards and reported the p-value. Finally, the abstract summary diagram was created with Biorender.com.

### Institutional review board statement

The animal study protocol was approved by the Institutional Animal Care and Use Committee (IACUC) of Dartmouth College (protocol code 0000224, approval date 2/24/2020).

## Results

### Development of a murine model with human Fanconi Anemia-Acute Myeloid Leukemia (FA-AML) and non-FA-mutated AML

**Fig 1:**
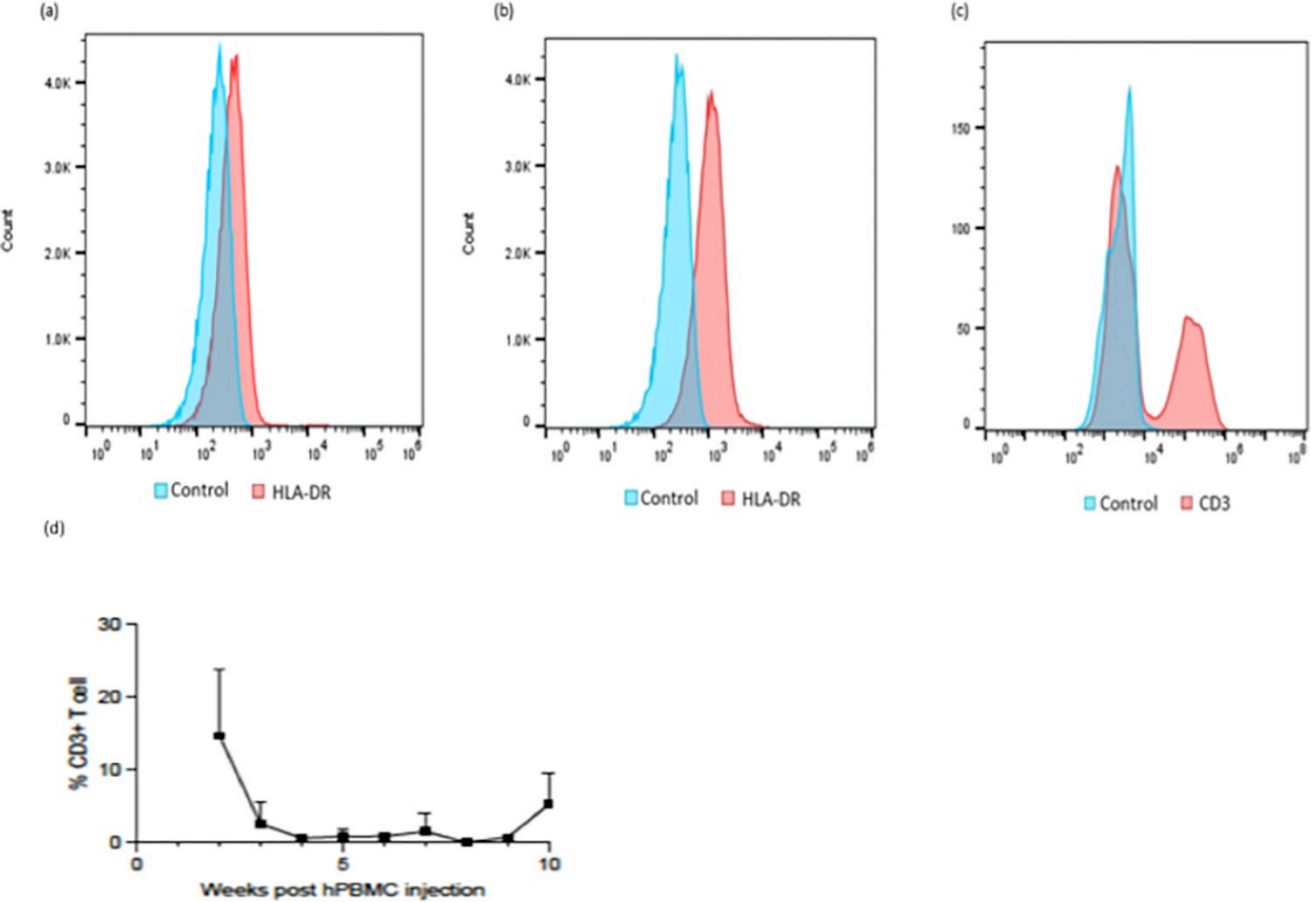
Development of a humanized murine AML model. FA-AML1 or Kasumi-1 cells were injected into 8–10 weeks old NSG mice. (a) Kasumi-1 (b) and FA-AML1 engraftment was confirmed by flow cytometric analysis of peripheral blood for HLA-DR expression (compared to unstained controls). Human PBMCs were injected into mice one week after leukemia cell engraftment. (c) Engraftment of human PBMCs was done by flow cytometric analysis for CD3 expression. (d) Persistence of human T cell characterized by CD3+ in blood in FA-AML1 engrafted mice (n = 6). Human PMBCs were injected after confirmation of HLA-DR positivity and monitored for human CD3 expression weekly for 15 weeks post-hPBMC injection.

Here, we developed a novel humanized murine model involving co-transplantation of both human AML and human immune cells to investigate the effectiveness of new immunotherapeutic treatments. Successful AML (FA-AML1 or Kasumi-1) engraftment in NSG mice was confirmed by positive HLA-DR expression (compared to unstained controls) in peripheral blood by flow cytometry 8–14 weeks post leukemic cell injection (Fig 1A and 1B). Engraftment time was faster in FA-AML1, and anti-human HLA-DR staining showed no cross-reactivity with murine cells in whole blood, so HLA-DR is a marker for human cell engraftment (S1 Fig). We reconstituted a human immune system following engraftment in the mice with an injection of allogeneic human peripheral blood mononuclear cells (hPBMCs), which was confirmed through flow cytometry by the presence of CD3^+^ T cells (Fig 1C). Gating strategies for the flow cytometric analyses for Fig 1A–1C are included in S2 Fig. Treatment was initiated 7 days after hPBMC engraftment confirmation with intraperitoneal injection of Nivolumab (PD-1 inhibitor) or PBS twice a week, and tumorigenic endpoints were obtained. Nivolumab does not have anti-leukemic activity *in vitro* (S3 Fig). Persistence of human CD3^+^ T cells in engrafted mice was observed up to 15 weeks post hPBMC injection (Fig 1D). Engraftment of hPBMCs did not cause significant body weight changes (S4 Fig).

### Higher PD-L1 expression in FA-AML1 engrafted mice correlates with greater response to PD-1 blockade

**Fig 2:**
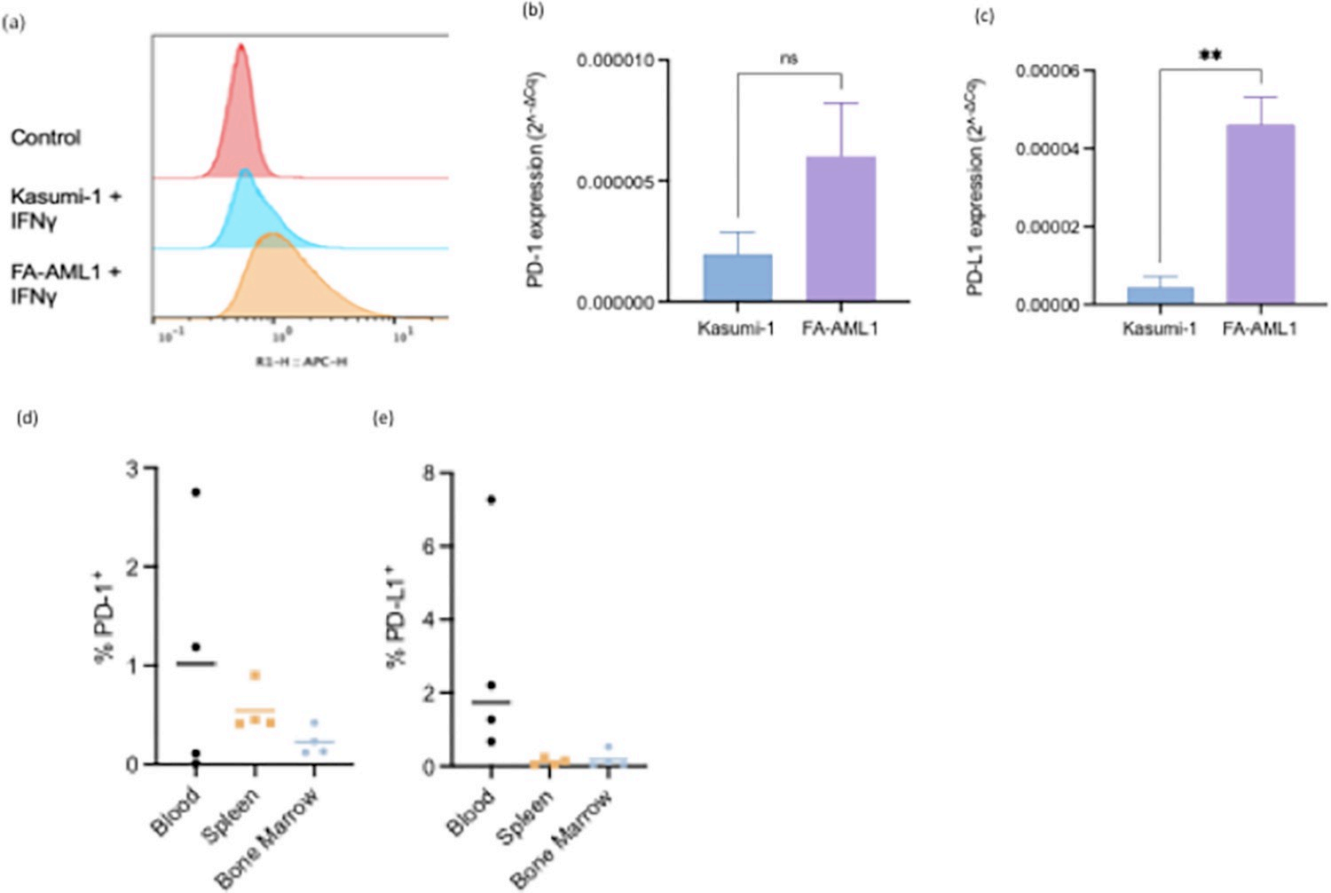
*In vitro* and *in vivo* expression of PD-1 and PD-L1. (a) PD-1 and (b) PD-L1 mRNA expression in Kasumi-1 and FA-AML1 cells stimulated by 10 ng/mL IFNγ for 24 hours. (c) PD-1 and (d) PD-L1 expression in blood, spleen and bone marrow of FA-AML1 engrafted mice.

It has been previously reported that PD-L1 expression may serve as a predictive biomarker for anti-PD-1 response across various cancer types [20–23]. We first examined PD-1 and PD-L1 expression in FA-AML1 and Kasumi-1 cells in an attempt to stratify Nivolumab response based on FA/BRCA mutational status. FA-AML1 cells expressed higher levels of PD-1 and PD-L1 compared to Kasumi-1 under IFNγ stimulation, mimicking the *in vivo* increased inflammatory cytokine response observed in FA patients (Fig 2A–2C) [24]. PD-1 and PD-L1 expression was also confirmed in engrafted mice (Fig 2D and 2E).

**Fig 3:**
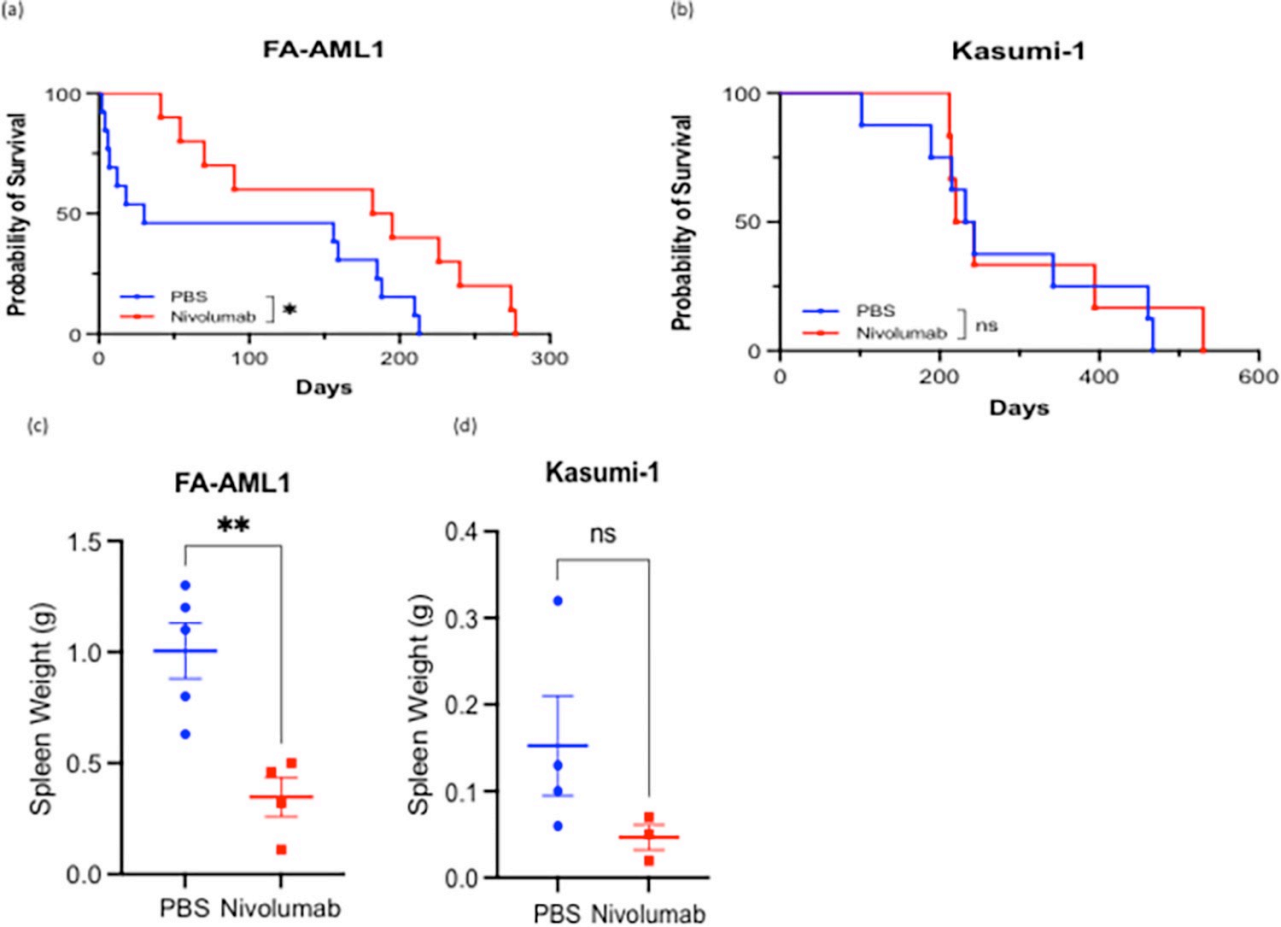
Response to Nivolumab is stratified by PD-L1 expression in AML-engrafted humanized mice. (a) Overall survival curves of mice engrafted with either FA-AML-1 (cells with high PD-L1 expression) (PBS: n = 13, Nivolumab: n = 12) and (b) Kasumi-1 (cells with low PD-L1 expression) (PBS: n = 8, Nivolumab: n = 8). Spleen weights of mice that are PBS treated versus Nivolumab treated in (c) FA-AML-1 engrafted (PBS: n = 5, Nivolumab: n = 4) and (d) Kasumi-1 engrafted (PBS: n = 4, Nivolumab: n = 3) mice.

**Fig 4:**
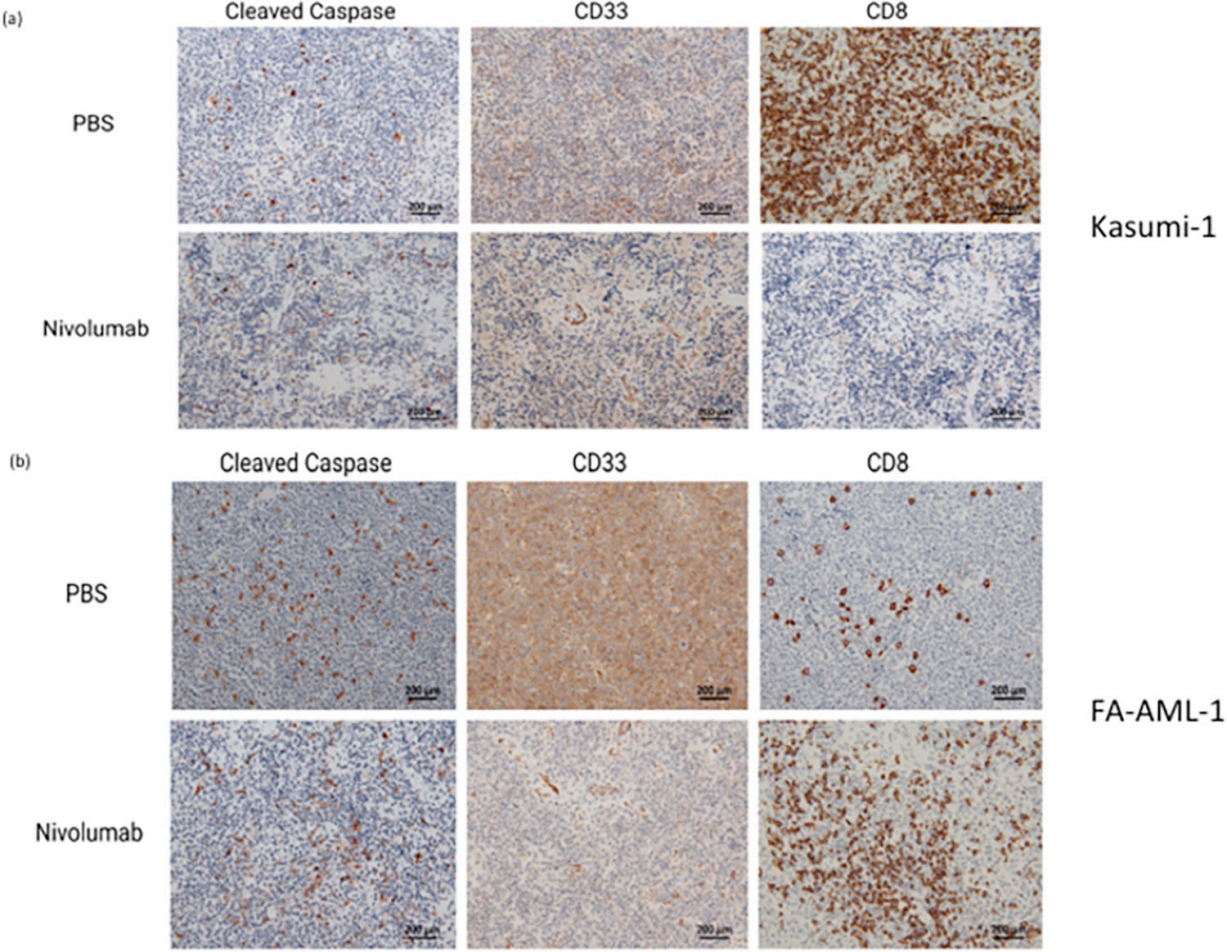
Immunohistochemical detection of cleaved Caspase 3, CD33 and CD8. Spleens of (a) Kasumi-1 and (b) FA-AML1 engrafted mice treated with PBS or Nivolumab.

Based on PD-L1 expression, it is hypothesized that FA-AML1 engrafted mice will respond better to Nivolumab compared to Kasumi-1 engrafted mice. Overall survival was used as an endpoint to investigate the effectiveness of PD-1 blockade on FA-AML1 and Kasumi-1 engrafted mice. Overall survival was significantly increased with Nivolumab treatment in FA-AML1 engrafted mice (p = 0.0216) but not Kasumi-1 engrafted mice (Fig 3A and 3B). Kasumi-1-engrafted mice survived longer regardless of treatment compared to FA-AML1-engrafted mice, likely due to the increased aggressiveness of FA-AML compared to non-FA-mutated AML. Since the spleen is a common site of leukemic infiltration, we evaluated spleen weights after Nivolumab or PBS treatment in FA-AML1 and Kasumi-1 engrafted mice. Spleen weights were significantly decreased after Nivolumab treatment in both FA-AML1 and Kasumi-1 engrafted mice compare to treatment naïve mice (Fig 3C and 3D). We also observed a decrease in CD33^+^leukemic blasts in the spleens of Nivolumab-treated mice, suggesting that Nivolumab effectively decreases leukemic burden in the spleen (Fig 4, S5 Fig). Response to Nivolumab in FA-AML1 engrafted mice was accompanied with an increase in CD8^+^ T cell infiltration and subsequent apoptosis with increased cleaved Caspase-3 in the spleen (Fig 4). Our observation of FA/BRCA deficient cancer cells having higher PD-L1 expression has also been reported in BRCA-deficient ovarian and breast cancer and considered a biomarker of response to anti-PD1 therapy [25, 26].

### Leukemic infiltration into organs in both FA-AML1 and Kasumi-1 engrafted mice

Most types of leukemia show various degrees of infiltration into other organs, most commonly in the spleen, liver, lungs and kidneys. It is important that our mouse model recapitulate the leukemic infiltration observed clinically in order to accurately represent human AML *in vivo*. Histopathological analyses of vital organs from engrafted mice show both FA-AML1 and Kasumi-1 infiltrated into spleen, liver, lungs and kidneys (S5A and S5B Fig). Histopathology for AML in the organ sections was identified for blast morphology (increased nuclear:cytoplasmic ratio and dense chromatin, increased nuclear blue stain). There is more infiltration of FA-AML1 leukemia cells than of Kasumi-1 leukemia cells, likely reflecting the more aggressive nature of FA-AML1 leukemia. Treatment naive mice had significantly more AML blasts in the organs compared to Nivolumab-treated mice in both FA-AML1 and Kasumi-1 engrafted mice. Of note, lung histology shows signs of inflammation with increased cells present in alveolar spaces and loss of tissue architecture in only Nivolumab-treated mice in FA-AML1 engrafted mice, which are consistent with previously reported ICI-related pulmonary toxicities [27]. However there is a lack of signs of GVHD (no gross skin changes, no increased lymphocytic infiltrate, no loss of tissue architecture or apoptotic blebbing). Thus, the engrafted FA-AML1 and Kasumi-1 in this model replicates human AML disease progression with leukemic infiltration into vital organs, with low levels of toxicity.

## Discussion

The ability to study human tumor cells *in vivo* advances murine modeling of diseases, and advances the treatment of human tumors using immunotherapies [6, 28]. Humanized murine models of AML have included xenografts of cell lines or patient samples [29]. In some publications, there is successful engraftment of a humanized immune system with bone marrow transplantation using CD34+ hematopoietic stem cells, or with peripheral blood mononuclear cells (PBMCs). To better study FA-AML biology, a group has engrafted human FA-AML cells into mice, but without a humanized immune system. We are the first to engraft human FA-AML cells and a humanized immune system in order to test the anti-PD-1 immune checkpoint inhibitor Nivolumab in both FA-mutated AML and non-FA mutated AML. Our humanized mice allow for the study of naturally occurring AML in FA patients, as opposed to previous models of chemically or radiation-induced AML in transgenic mice with germline FA mutations. We were able to successfully engraft both Kasumi-1 and FA-AML1 human leukemia cells into NSG mice. Engraftment occurred faster with FA-AML1 cells than with Kasumi-1 cells, which may reflect the relative aggressiveness of FA-AML1, which is a relapsed FA-AML, compared to the primary Kasumi-1 AML. Other factors that can affect engraftment of human tumor cells in mice include the presence of human cytokines, therefore NSG-SGM3, with transgenic expression of human IL3, GM-CSF and SCF, may also be used to expedite AML engraftment in our humanized murine model [30].

After confirmation of leukemic engraftment, we engrafted immune cells and reconstituted a human immune system in these mice using hPBMCs [31]. We are the first to report such a humanized murine model for FA-mutated AML with immune cells. With engraftment of allogeneic human PBMCs, we find an anti-leukemic effect, i.e. decrease in percent of leukemia cells in the peripheral blood. Human PBMCs in NSG mice can cause graft-versus-host disease (GvHD) within 6 weeks of hPBMC engraftment [31]. We did not observe signs of GvHD histopathologically or grossly in the time we used our model, except for two mice that had hair loss and decreased overall survival, a possible sign of skin GvHD [32]. An alternative strategy to humanize mice with immune cells would be to perform bone marrow transplantation with CD34^+^ human hematopoietic stem cells. However, this strategy is more time-consuming and requires bone marrow conditioning with chemotherapy or irradiation, although GvHD is less likely to occur than with hPBMCs [33, 34]. Therefore, our model has the advantage of efficient engraftment without conditioning and can be used for short term studies.

We were able to functionally test our humanized murine model with ICI immunotherapy. Nivolumab treatment resulted in significantly decreased spleen weight and leukemic infiltration into organs in both Kasumi-1 and FA-AML1 engrafted mice with humanized immune systems. We hypothesized that AML with a DNA damage repair defect, i.e. FA-mutated, would respond more effectively to ICI immunotherapy than non-FA-mutated AML, similarly to previous success of ICI in mismatch repair-deficient colon cancer [35, 36]. Nivolumab-treated FA-AML1-engrafted mice, but not Kasumi-1-engrafted mice, had prolonged overall survival. Higher PD-L1 expression in FA-AML1 may also account for prolonged overall survival in FA-AML1 engrafted mice, as patients with higher PD-L1 expression have higher response rates to anti-PD-1/PD-L1 therapy [37, 38]. We also observed increased apoptosis and CD8^+^ T cell infiltration in Nivolumab-treated FA-AML1 engrafted mice. However, it is unknown why there is some anti-leukemic activity of Nivolumab on non-FA-mutated AML [39, 40], nor do we know the mechanism behind the greater efficacy of nivolumab against FA-mutated AML, but investigation into these characteristics is beyond the scope of this work and planned for future study.

ICI immunotherapy has a side effect and toxicity profile consisting mostly of inflammation of the organs such as pneumonitis and colitis [41]. In our humanized murine model, the mice did not have evidence of these toxicities. The mice had normal respiration, normal bowel movements, normal motor capabilities and no significant weight changes. Therefore, Nivolumab is generally well tolerated in this murine model of humanized FA-AML. However, histopathology of the organs showed evidence of organ inflammation in the Nivolumab-treated cohort, but not in the treatment-naïve cohort. The histologic inflammation of the organs can be reversed after discontinuation of ICI immunotherapy [42]. However, it is possible that the Nivolumab treated mice died due to ICI-related toxicity.

In summary, we developed a novel humanized murine model for studying FA-mutated AML, and evaluated the effectiveness of Nivolumab in successfully engrafted mice. We found that Nivolumab has higher anti-leukemic activity in FA-mutated AML than non-FA-mutated AML with minimal gross toxicities. This humanized murine model can be further optimized to improve time to engraftment, ability to engraft primary human AML samples, profiling of immune cell subtypes over time, and minimizing graft-versus-host disease. As a model to study ICI immunotherapy, future studies can be done to further characterize the mechanism of anti-leukemic effects of ICIs, and how to minimize ICI-related toxicities.

## Conclusions

This novel murine model illustrates that humanizing NSG mice with human AML followed by immune cells from hPBMCs can be used to study the effectiveness of immune checkpoint inhibitors. Specifically, we were able to engraft FA-mutated AML and non-FA-mutated AML, followed by engraftment of human immune cells, in order to test the anti-leukemic effects of ICI. We found PD-1 inhibition with Nivolumab significantly improved overall survival of mice with FA-mutated AML, but not of mice with non-FA-mutated AML. This humanized murine model is a platform for future study of cancers arising from Fanconi Anemia and testing of cancer immunotherapies.

## Supporting information

S1 Fig (a) Engraftment time of Kasumi-1 and FA-AML1 in NSG mice, as defined by HLA-DR positivity in the peripheral blood. (b) There is no cross reactivity of HLA-DR expression in murine whole blood. In contrast, both human AML cell lines Kasumi-1 and FA-AML1 are positive for HLA-DR, the marker used to determine AML cell engraftment.
(TIF)

S2 Fig Gating strategies for Fig 1A–1C. Single cells are gated on the lymphocytic population identified by SSC-H/FSC-H. HLA-DR+ and CD3+ populations are further gated on single cells based on compensation beads.
(TIF)

S3 Fig No change in cell viability of (a) FA-AML1 and (b) Kasumi-1 cell lines in vitro under 48 hours of Nivolumab treatment in varying concentrations.
(TIF)

S4 Fig NSG mice engrafted with hPBMCs do not show significant body weight changes up to 10 weeks post hPBMC injection. (TIF)

S5 Fig Histopathological analyses of mouse vital organs (spleen, kidney, lungs and liver). Representative images of H&E stained sections of organs from (a) FA-AML1 and (b) Kasumi-1 engrafted mice treated with PBS or Nivolumab. (scale bar = 200microns).
(TIF)
