# Supplementary figures and images for: A murine model to evaluate immunotherapy effectiveness for human Fanconi anemia-mutated acute myeloid leukemia
Source: PLoS One. 2024 Jan 30;19(1):e0292375. doi: 10.1371/journal.pone.0292375 (PMC10826936; doi:10.1371/journal.pone.0292375)

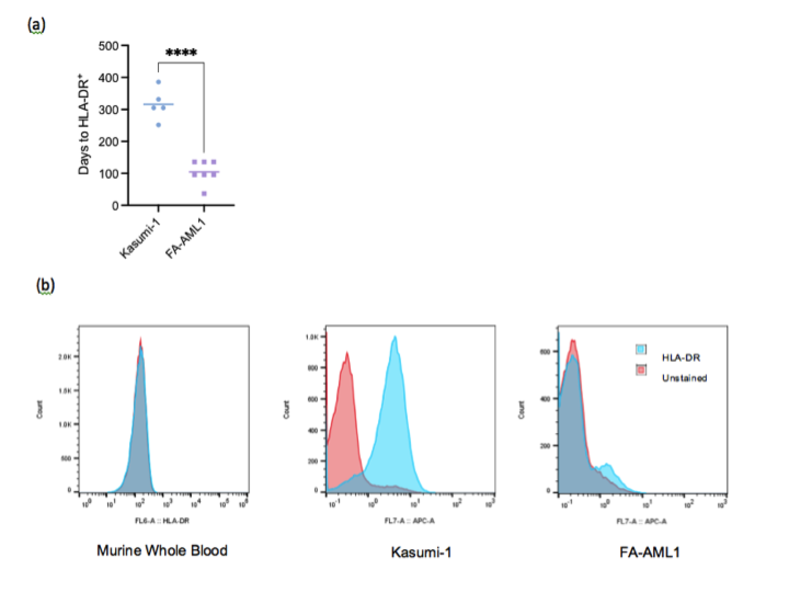

Supplement: S1 Fig — (a) Engraftment time of Kasumi-1 and FA-AML1 in NSG mice, as defined by HLA-DR positivity in the peripheral blood. (b) There is no cross reactivity of HLA-DR expression in murine whole blood. In contrast, both human AML cell lines Kasumi-1 and FA-AML1 are positive for HLA-DR, the marker used to determine AML cell engraftment. (TIF) [file pone.0292375.s001.tif]

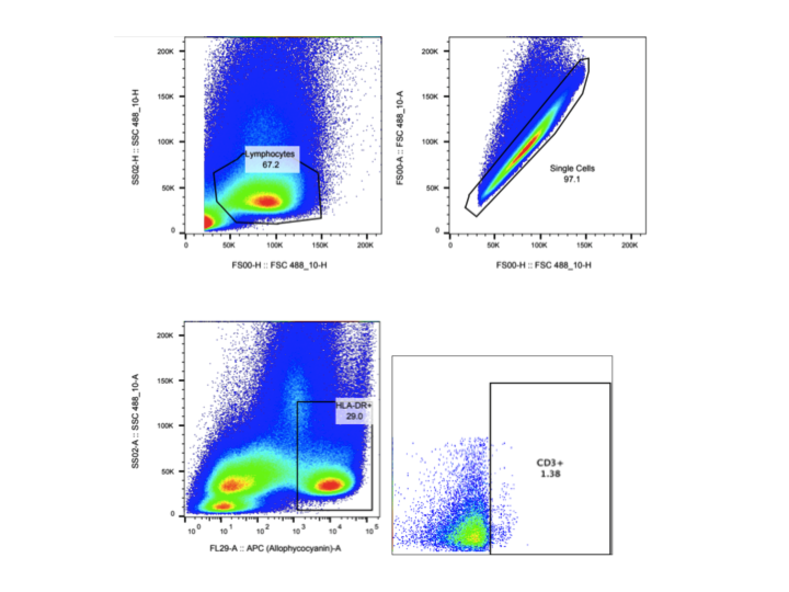

Supplement: S2 Fig — Single cells are gated on the lymphocytic population identified by SSC-H/FSC-H. HLA-DR+ and CD3+ populations are further gated on single cells based on compensation beads. (TIF) [file pone.0292375.s002.tif]

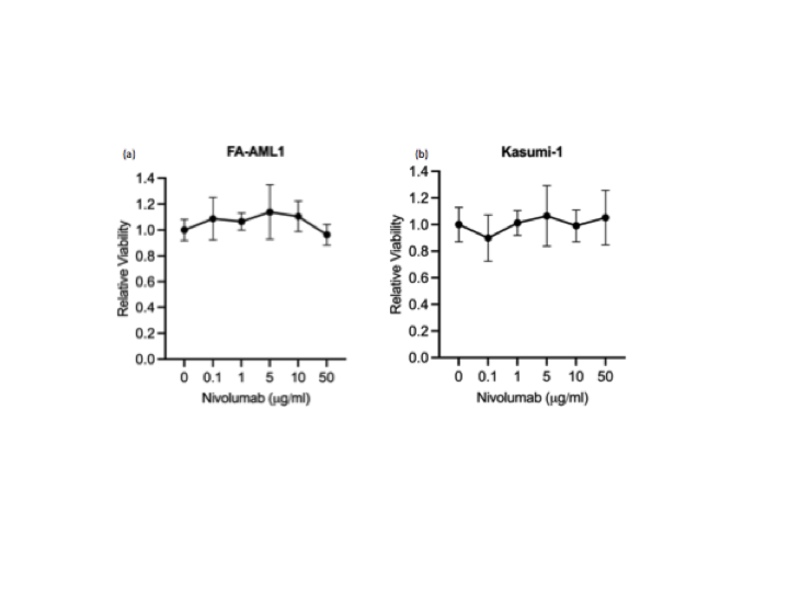

Supplement: S3 Fig — No change in cell viability of (a) FA-AML1 and (b) Kasumi-1 cell lines in vitro under 48 hours of Nivolumab treatment in varying concentrations. (TIF) [file pone.0292375.s003.tif]

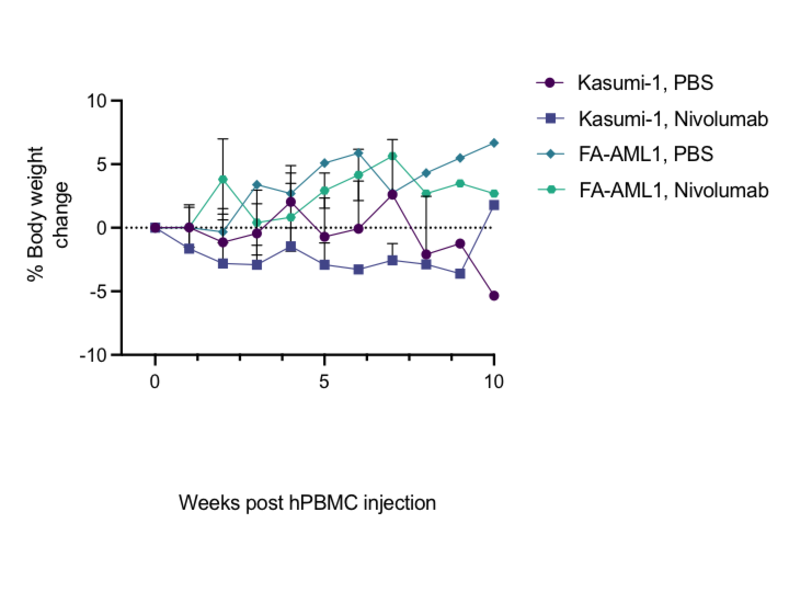

Supplement: S4 Fig — (TIF) [file pone.0292375.s004.tif]

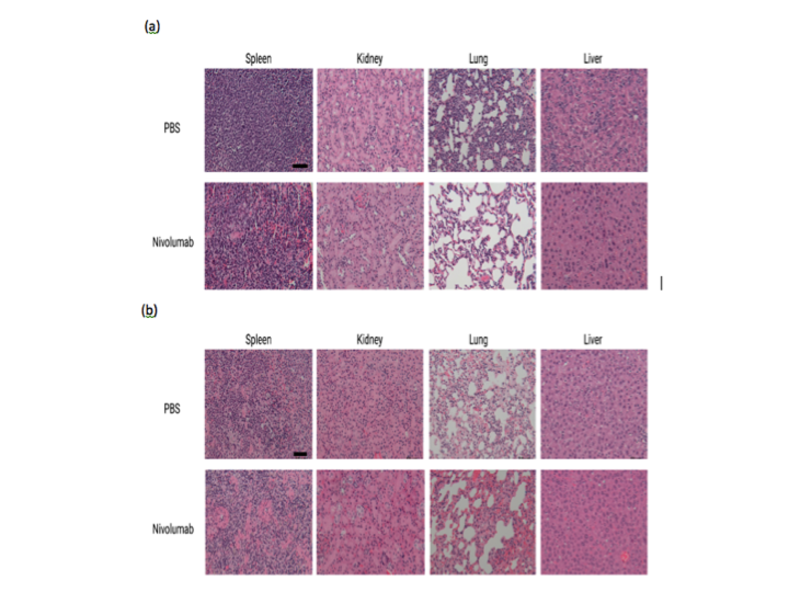

Supplement: S5 Fig — Representative images of H&E stained sections of organs from (a) FA-AML1 and (b) Kasumi-1 engrafted mice treated with PBS or Nivolumab. (scale bar = 200microns). (TIF) [file pone.0292375.s005.tif]
